# Noma disease (cancrum oris, orofacial gangrene) in an acute myeloid leukemia patient: a case report

**DOI:** 10.1186/s13256-022-03317-7

**Published:** 2022-03-08

**Authors:** Jiho Park, Sanghun Kim, Seung-Woo Shin, Chuhl Joo Lyu, Dongwook Kim

**Affiliations:** 1 Department of Oral & Maxillofacial Surgery, College of Dentistry, Yonsei University, Seoul, Korea; 2 Department of Pediatrics, College of Medicine, Yonsei University, Seoul, Korea

**Keywords:** Noma, Cancrum oris, Orofacial gangrene, Acute myeloid leukemia, Facial disfigurement

## Abstract

**Background:**

Noma is a rare disease that occurs mainly in malnourished patients in developing countries. Noma starts as facial swelling and gingival necrosis that eventually necrotizes underlying tissues including the jaw bone, leaving severe disfigurement. It is reported extremely rarely in patients with severe immunosuppression or blood dyscrasia.

**Case presentation:**

The gingivitis that occurred in a 12-year-old Asian female patient with acute myeloid leukemia was getting increasingly worse. Although the proper treatment was done, the patient’s condition did not improve, and eventually, a large full-thickness defect was left in the maxillofacial part.

**Conclusions:**

Early diagnosis and management is the only way to prevent the progression, which leads to facial disfigurement. We present a case of noma in a pediatric acute myeloid leukemia patient, in which oral function was restored through surgical intervention.

## Introduction

Noma (orofacial gangrene, cancrum oris) is a cryptogenic disease that leads to severe orofacial destruction [1]. Starting as gingivitis, noma develops into gingival necrosis that spreads to the underlying tissues, eventually involving the jawbone. The corresponding facial region develops swelling and subsequently necrotizes, leading to the destruction of soft and hard facial tissues to leave severe disfigurement.

Noma is known to occur mainly in patients with the following conditions: extreme poverty, severe malnutrition, unsafe drinking water, poor sanitation, poor oral health practices, high infant mortality, limited access to high-quality health care, and intrauterine growth retardation; usually in developing countries, but other cases are also reported extremely rarely, including those with human deficiency virus (HIV) infection, cyclic neutropenia, leukemia, Down’s syndrome, Burkett’s disease, and herpetic stomatitis [2–4].

In this study, we report 12-year-old female who suffered leukemia with a complication of noma disease on her lower face.

## Case report

**Fig. 1:**
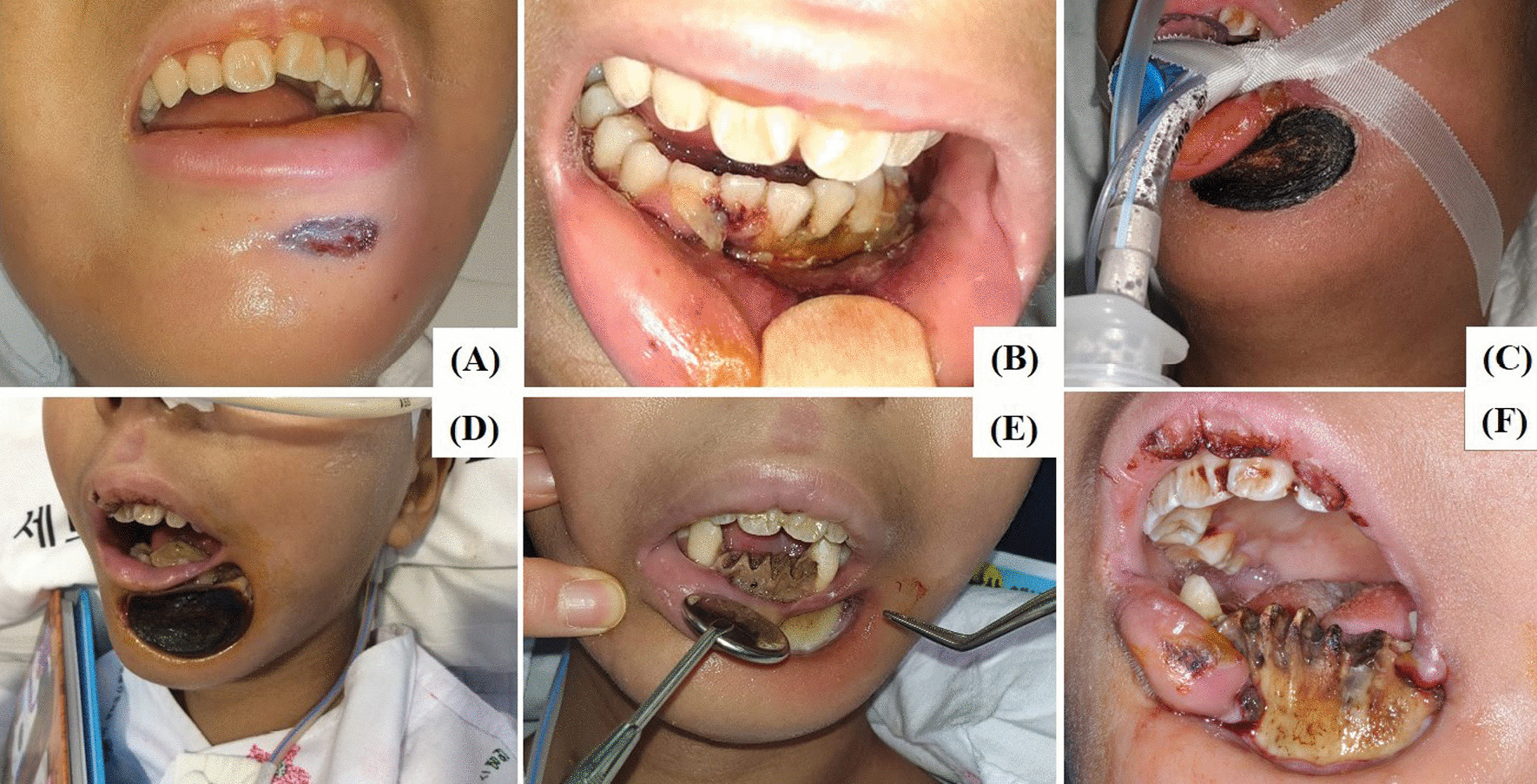
Initial presentation and progression of lesion. **A** and **B** Ecchymosis of chin and necrotizing gingivitis is noted. **C** 1 week, **D** 3 weeks, **E** 5 weeks, and **F** 7 weeks after the initial presentation. The necrotic portion of mandible and the alveolar bone spontaneously fell off afterwards

A 12-year-old Asian female patient was referred from the pediatric hematology-oncology service, department of pediatrics, for the evaluation and management of swollen gingiva and chin (Fig. 1). The patient had a history of patent ductus arteriosus (PDA) closure at age 6. She underwent four cycles of induction chemotherapy for acute myeloid leukemia (AML), which had been diagnosed a year previously. She was admitted for the management of neutropenic fever over 39 °C. She was on antibiotics (linezolid, meropenem, sulfamethoxazole, metronidazole, teicoplanin, cefpiramide, amikacin), antifungals (fluconazole, nystatin, amphotericin B, voriconazole, caspofungin), and an antiviral (acyclovir) for her febrile condition, under the impression of neutropenic fever and vancomycin-resistance enterococci (VRE) sepsis. The results of complete blood count with differential were as follows: white blood cell (WBC) counts 40/μL, platelet counts 8000/μL, absolute neutrophil count 0/μL.

As the patient was already on every possible antibiotic, antifungal, and antiviral, close observation and oral hygiene maintenance was the only possible management. Surgical intervention was contraindicated due to her systemic condition, especially considering her hematologic status. Six days after the initial presentation, the patient developed cardiac arrest under the background of uncontrolled fever. Return of spontaneous circulation was achieved after resuscitation, and her general condition improved as days went by. The oral and facial lesion progressed to full-thickness gangrene and underlying alveolar bone separated from the surrounding mandible (Fig. 1). Teeth on the segment spontaneously fell out (Fig. 1). The necrotic alveolar bone segment also spontaneously fell out.

**Fig. 2:**
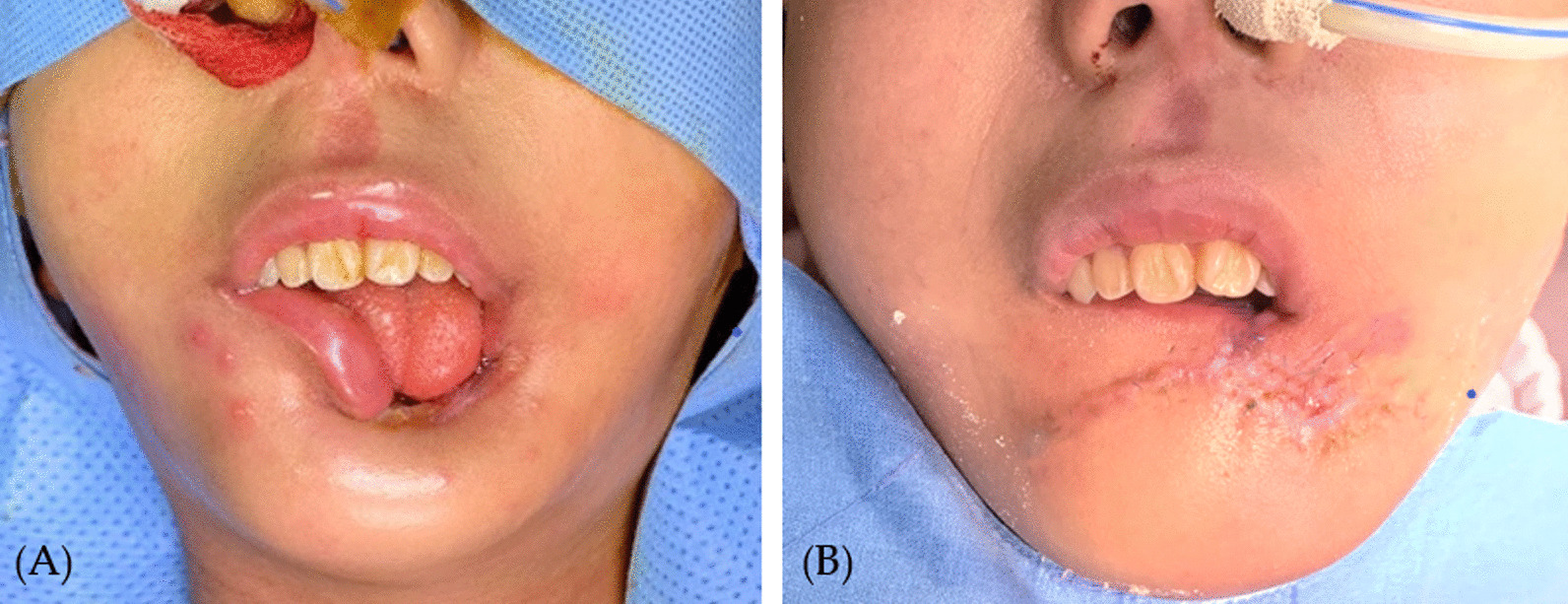
Preoperative and postoperative appearance

After the patient had become afebrile and able to return to regular activities, not only the disfigurement but the incompetency of the lips and resultant drooling posed severe obstacles in her daily life since oral intake was disabled. Considering the pancytopenic condition of the patient, surgical repair was done as simply as possible, by local flap, to achieve continuity of the orbicularis oris muscle and competency of the lip, thus enabling oral intake afterwards (Fig. 2).

## Discussion and conclusions

Noma was first described in 1595 by Carolus Battus, and since then many cases have been reported [5]. Following advances in medical science, novel epidemiological investigations and surgeries on noma were conducted in the 1800s [6]. In 1926, Smith *et al*. presented the first case of cancrum oris in leukemia patients after a postmortem examination of a patient who died of hospitalization due to severe pain in the left facial area. In 2010, Krishnamurthy *et al*. reported a case of cancrum oris in a patient with chemotherapy for acute myeloid leukemia [7, 8].

Annually, the incidence and prevalence of noma is reported as 140,000 and 770,000 cases, respectively. The epidemiology of noma has not changed much over the years, except that there has been a reduction in the mortality rate from 90% to about 8–10%, mainly due to modern antibiotics [9, 10]. Early diagnosis and management are the only way to prevent progression of the disease and resulting severe facial disfigurement.

Based on World Health Organization guidelines for noma in 2016, the optimal standard of care is to start antibiotic treatment as early as possible [4]. Although the patient in this report was already undergoing treatment with a wide range of antibiotic, antifungal, and antiviral drugs for the management of neutropenic fever, the lesion progressed and resulted in full-thickness defects including the underlying mandible.

Defects in the lower face can occur for various reasons, such as trauma and malignant tumors, and if accompanied by lip incompetency and drooling, greatly affect daily life. Repairing such defects will help enable oral intake and enhance appearance as well. Though there are various methods, including local and free flaps, to repair or reconstruct the lip and lower facial defects, a minimally invasive approach should be considered in such a pancytopenic patient. Flipping the buccal skin flap to repair the oral side and recovering the continuity of the orbicularis oris muscle was the key step of the repair.

With advances in medicine and living standards, noma has come to be regarded as a region-specific disease. It was thus initially difficult to achieve proper diagnosis of its occurrence in an acute myeloid leukemia patient as reported here. We report this case of noma in a child with leukemia to emphasize that this rare disease is not confined to the malnourished, but may also occur in patients with deliberately suppressed immune systems [11].

## Data Availability

Not applicable.

## Abbreviations

AML: Acute myeloid leukemia
VRE: Vancomycin-resistance enterococci
WBC: White blood cell
